# The effects of Fe, Mg, and Pt-doping on the improvement of Ni stabilized on Al_2_O_3_-CeO_3_ catalysts for methane dry reforming†

**DOI:** 10.1039/d3ra04809h

**Published:** 2023-11-09

**Authors:** Abbas Jawad

**Affiliations:** a Department of Chemical & Biochemical Engineering, Missouri University of Science and Technology 1101 N. State Street Rolla Missouri 65409 USA ajd5d@mst.edu abbasajd5d@gmail.com; b Midland Refineries Company MRC, AL Daura Refinery Company, Services Energy Board Baghdad Iraq

## Abstract

Herein, the promotional effects of Mg, Fe, and Pt on Ni-based catalysts supported on Al_2_O_3_-CeO_2_ (Ni/Al_2_O_3_-CeO_2_) were investigated in the dry reforming of methane (DRM) reaction. The interaction of a suitable amount of MgO and FeO with Ce_2_O_3_ stabilized in the catalysts was demonstrated by the temperature-programmed desorption of CO_2_ (CO_2_-TPD). Ce_2_O_3_ has a high basicity for adsorbing CO_2_, generating a monoclinic Ce_2_O_2_CO_3_ species in the DRM reaction. Surface oxygen ions were also produced by adding MgO and FeO, as demonstrated by the temperature-programmed reduction of H_2_ (H_2_-TPR). Monoclinic Ce_2_O_2_CO_3_ and surface oxygen may both be used to oxidize and remove the carbon that was deposited, maintaining the high activity and stability of the metal Ni and Pt catalysts. The high dispersion and synergistic interactions between the platinum and oxide phases, which are associated with the decrease in reduction temperature and the rise in the number of basic sites, are responsible for the increased activity of Pt with M–Ni/Al_2_O_3_-CeO_2_ catalysts. The co-doped Ni/Al_2_O_3_-CeO_2_ catalysts with Mg and Fe significantly enhanced the activity (more than 80% methane and 84% CO_2_ conversion), the selectivity toward syngas (∼90%), and maintained the H_2_/CO ratio at about 0.97 at 700 °C.

## Abstract 2

Herein, the promotional effects of Mg, Fe, and Pt on Ni-based catalysts supported on Al_2_O_3_-CeO_2_ (Ni/Al_2_O_3_-CeO_2_) were investigated in the dry reforming of methane (DRM) reaction.

## Graphical abstract

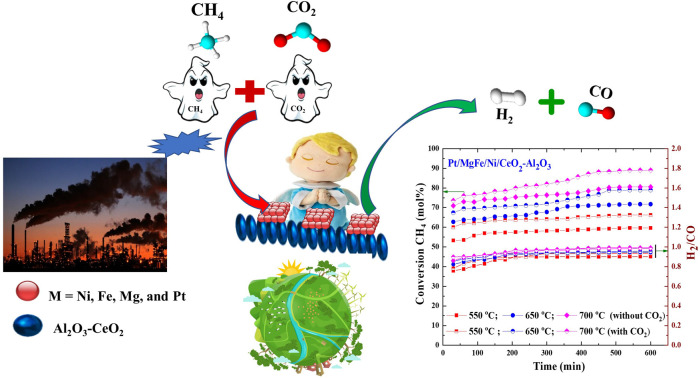

## 1. Introduction

The dry reforming of methane (DRM) with CO_2_ has attracted attention because of the utilization of two major greenhouse gases (CH_4_ and CO_2_) as feedstocks and provides a route for converting them into the low H_2_/CO ratio syngas, which can be directly used as fuel or to produce chemicals and fuels by the methanol synthesis and Fischer–Tropsch (FT) processes.^1–3^ DRM is an endothermic reaction and is usually conducted at a very high operation temperature (>800 °C) to ensure high methane conversion and minimize carbon deposition thermodynamically.^4,5^

The majority of the catalysts investigated for DRM are generally made up of group VIII transition metals, such as Ni due to their high activity.^6,7^ Promoting nickel-based catalysts with various metals, such as Mg, Fe, Zr, Cr, Ce, V, Mo, Rh, Pt, Pd, and Ru, is the most widely practiced approach for modifying DRM catalysts.^7–10^ In particular Pt, Rh, and Ru are highly active towards DRM, which enhances the stability against coke deposition as compared to the other non-promoted nickel-based catalysts.^11,12^ The catalyst performance is dependent on the Ni/metal ratio and the nature of the support.^6,13,14^ Both the promoters and support play important roles in metal electron transfer, cluster stabilization, and reducibility.^15^ In particular, it has been shown that Ni catalysts are highly reducible in the presence of noble metals, which enables both methane combustion and reforming to occur simultaneously, thereby resulting in higher energy efficiency and improved catalytic activity.^4^ Although noble metals are much more resistant to carbon deposition than other metal-based catalysts, they are generally uneconomical. Developing bimetallic catalysts by combining nickel with other metals is an alternative route to obtaining highly coke-resistant Ni-based catalysts for the DRM reaction.^16^ Several studies have been dedicated to improving the Ni-based CeO_2_-Al_2_O_3_ performance and stability through the addition of second metal promoters, such as Co, Pd, and Pt. It has been confirmed that adding a trace of transition metals can modify Ni surface properties by promoting the reducibility of Ni and thus increasing the number of active sites to achieve better catalytic performance.^9,17^ For example, several bimetallic catalysts such as Ni–Co, Ni–Pd and Ni–Pt with different supports (*e.g.*, SiO_2_, Al_2_O_3_, CeO_2_, MgO, TiO_2_, ZrO_2_, H-ZSM-5) have exhibited much higher activity and carbon resistance than monometallic Ni catalysts.^10,18–20^

Developing bi/trimetallic catalysts is a viable and critical method for developing highly coke-resistant Ni-based DRM catalysts, and there has been tremendous research interest in this regard in recent years. Combining nickel with other metals can easily change its surface properties, resulting in improved catalytic activity; this phenomenon is known as the synergistic impact.^21^ Noble metal catalysts such as Pt, Ru, and Rh exhibit good performance and selectivity for DRM reactions, even though noble metals have a high stability to carbon agglomeration at high temperatures.^6^ However, due to their high cost and scarcity, they are not economically competitive with other transition metal-based materials. Elsayed *et al.*^22^ discovered that supported bimetallic catalysts have good activity and stable DRM reaction capabilities. To assess the stability, platinum (0.2–2 wt%), nickel (8 wt%), and magnesium (8 wt%) were immobilized onto a ceria-zirconia support. Precipitation was used to create ceria–zirconia (0.6 : 0.4) solid solutions, and the metals were loaded using the incipient wetness method. The combination of Pt with NiMg/(Ce,Zr)O_2_ catalysts improved low-temperature dry reforming activity when compared to the control catalysts without Ni, Mg, and Pt. Among the various supports investigated, Al_2_O_3_ and HZSM-5 possessed greater surface areas, which enhanced the Ni particle dispersion; nevertheless, their inherent acidity and the greater interactions between the metal and support led to the rapid deactivation of the catalyst due to severe coke deposition. With the excellent thermal stability of CeO_2_ and the high specific surface area of Al_2_O_3_, which promotes the binding of tiny metal nanoparticles while also promoting CO_2_ binding in the catalytic processes by generating flexible carbonate-like species,^23^ a combined framework of these two materials was proposed and showed enhanced catalytic performance.^7,24,25^ In particular, cerium oxide has been widely investigated by many researchers as a promoter and support for nickel-based catalysts due to its unique redox properties (Ce^4+^/Ce^3+^) and remarkable oxygen storage capacities for the DRM reaction.^26^ Fang *et al.*^27^ investigated the impact of the promotors (Ce, Zr, and Al) employed to promote Y_2_O_3_ as supports for Ni/NiO to obtain more efficient catalysts for the DRM process. The XRD and Raman data showed that all three cations were doped into the Y_2_O_3_ lattice to form a solid solution structure, resulting in supports with decreased crystallinity and enhanced surface areas. As a result, all the changed catalysts had increased reaction performance. The Ni-support interaction of the modified catalysts was improved as compared to the unmodified catalyst, resulting in improved Ni dispersion. Furthermore, the modified catalysts had increased alkalinity, which is advantageous for activating CO_2_ and increasing activity. Most of the published studies reported improvement in catalytic behavior, however, little is known about the nature, structure and performance of Ni–M/CeO_2_-Al_2_O_3_ catalysts, where M = Fe, Fe–Mg, and Fe–Mg–Pt.

The aim of this study is to improve the performance of Ni-based CeO_2_-Al_2_O_3_ composite catalysts by synthesizing and structurally characterizing a series of monometallic M–Ni (M = Fe), bimetallic M–Ni (M = Mg–Fe and Pt–Fe) and trimetallic M–Ni (M = Mg–Fe–Pt) supported on CeO_2_-Al_2_O_3_ to suppress coke formation and maintain the H_2_/CO ratio. The role of the Pt co-promoter in composite catalysts was studied in the DRM reaction to distinguish the catalytic effects of Pt. Among all the synthesized M–Ni/CeO_2_-Al_2_O_3_ catalysts, the Pt/MgFe/Ni/CeO_2_-Al_2_O_3_ catalyst has shown the best performance by enhancing the CH_4_ and CO_2_ conversion and selectivity toward syngas, as well as extending the catalyst life.

## 2. Experimental

### 2.1. Materials

#### 2.1.1 Reagents

The following compounds were utilized in the manufacture of doped Ni-based catalysts and the construction of composite catalysts. The support for CeO_2_-Al_2_O_3_ with a CeO_2_ concentration of 50% on Al_2_O_3_ (Sigma-Aldrich), and metal precursors such as Ni(NO_3_)_2_·6H_2_O, Fe(NO_3_)_3_·9H_2_O, and Mg(NO_3_)_2_·6H_2_O were bought from Sigma-Aldrich (99.99+). Airgas supplied ultra-high pressure (UHP) H_2_, CO_2_, and CH_4_ gases.

#### 2.1.2 Synthesis of the Al_2_O_3_ support

Aluminum hydroxide was precipitated from an aqueous Al(NO_3_)_3_ solution using ammonia as the precipitating agent. At a temperature of 80 °C, the precipitation process was carried out by adding ammonia to the nitrate solution until the pH of the solution changed from acidic to basic (pH = 9–10). The Al(OH)_3_ precipitate was aged for 24 h, then filtered and rinsed with deionized water until the solution's pH was 7. The precipitate was obtained, which was then dried at 100 °C for 1 h and calcined at 550 °C in an oxygen stream for 4 h.

Cerium(iii) nitrate hexahydrate was used to create the CeO_2_-Al_2_O_3_ support. The aqueous nitrate solution was added dropwise to the previously obtained alumina support, then, the resulting solution was aged for 24 h. After the solvent had evaporated, the solid residue was dried at 100 °C for 1 h before being calcined in air for 5 h at 550 °C. CeO_2_ was loaded to a 50% level.

#### 2.1.3 Synthesis of monometallic systems

The monometallic Ni catalyst was prepared using the incipient wetness impregnation method. An aqueous solution of nickel(ii) nitrate was used to deposit a nickel phase onto CeO_2_-Al_2_O_3_, which was then impregnated for 24 h. The catalyst was produced, dried at 100 °C, and then calcined at 550 °C for 5 h after the solvent had evaporated. The catalyst was obtained with a nominal metal concentration of 4%.

#### 2.1.4 Synthesis of bimetallic systems

The bimetallic catalyst Fe–Ni was obtained by subsequent impregnation using an aqueous Fe(NO_3_)_3_ solution. The impregnation process was the same as previously stated. The catalyst was obtained with a nominal Fe concentration of 2%.

#### 2.1.5 Synthesis of trimetallic systems

The trimetallic catalyst Mg–Fe–Ni was obtained by subsequent impregnation using an aqueous Mg(NO_3_)_2_ solution. The impregnation process was the same as previously stated. The catalyst was obtained with a nominal Fe concentration of 2%. The aqueous H_2_PtCl_6_·6H_2_O solution was impregnated into (Mg–Fe–Ni and Fe–Ni) catalysts. The impregnation procedure was similar to that reported before. The nominal Pt content in the obtained catalysts was 0.0005%. The metal loadings were set at 4 wt% nickel for Ni/CeO_2_-Al_2_O_3_; 4 wt% nickel, 2 wt% iron for Fe/Ni/CeO_2_-Al_2_O_3_; 4 wt% nickel, 2 wt% iron, and 0.005 wt% platinum for Pt/FeNi/CeO_2_-Al_2_O_3_; 4 wt% nickel, 2 wt% iron, and 0.5 for magnesium for MgFe/Ni/CeO_2_-Al_2_O_3_; 4 wt% nickel, 2 wt% iron, 0.5 for magnesium, and 0.005 wt% platinum for Pt/MgFe/Ni/CeO_2_-Al_2_O_3_.

### 2.2. Instrumental measurements

X-ray diffraction (XRD) patterns of the catalysts were obtained by a diffractometer using a PANalytical instrument operating at 30 kV and 15 mA. The XRD pattern was evaluated at a step size of 0.026° from 2*θ* = 5° to 90° and a rate of 2° min^−1^. N_2_ physisorption isotherm measurements were carried out in a Micromeritics 3Flex surface characterization analyzer at 77 K. Textural properties such as surface area, total pore volume, micropore volume, and average pore width were determined using Brunauer–Emmett–Teller (BET), Barrett–Joyner–Halenda (BJH), and *t*-plot methods, respectively. Prior to the measurements, samples were degassed at 250 °C for 6 h using a Smart VacPrep. H_2_-TPR measurements were carried out in a U-shaped quartz cell using a 5% vol H_2_/He gas with a flow rate of 50 cm^3^ min^−1^ at a heating rate of 10 °C min^−1^ up to 900 °C by using a Micromeritics 3Flex analyzer. Hydrogen consumption was followed by on-line mass spectroscopy (BELMass) and quantitative analysis was done by comparison of the reduction signal with the hydrogen consumption of a CuO reference. The temperature-programmed desorption of CO_2_ (CO_2_-TPD) was performed on the same Micromeritics 3Flex analyzer. Prior to adsorption measurement, all samples were initially reduced at a temperature of 200 °C in a 5% H_2_ in He gas mixture and held at the reduction temperature for 1 h, then cooled down to 50 °C under He. After the temperature was stabilized, the sample was exposed to 10% CO_2_ in He for 30 min. To remove physically bound CO_2_ from the surface, a flow of He (50 cm^3^ min^−1^) for 30 min at 50 °C was used. The desorption of CO_2_ was measured from 50 to 600 °C at a constant heating rate of 10 °C min^−1^. To determine the nature of surface acid sites, Fourier-transform infrared spectroscopy (FTIR) of pyridine, using a Bruker Tensor spectrophotometer, was employed to determine the types of acid sites present in the samples. All samples were activated at 450 °C for 4 h to release the moisture before the adsorption of pyridine and cooled down to 60 °C for pyridine adsorption until saturation. All the measured spectra were recalculated to a “normalized” wafer of 10 mg. For the quantitative characterization of acid sites, the bands at 1450 and 1550 cm^−1^ were considered to correspond to Lewis and Brønsted sites, respectively. Furthermore, inductively coupled plasma mass spectrometry (ICP-MS) analyses were used to obtain the chemical composition of the surfaces and bulk before reaction.

### 2.3. Catalytic tests

**Scheme 1:**
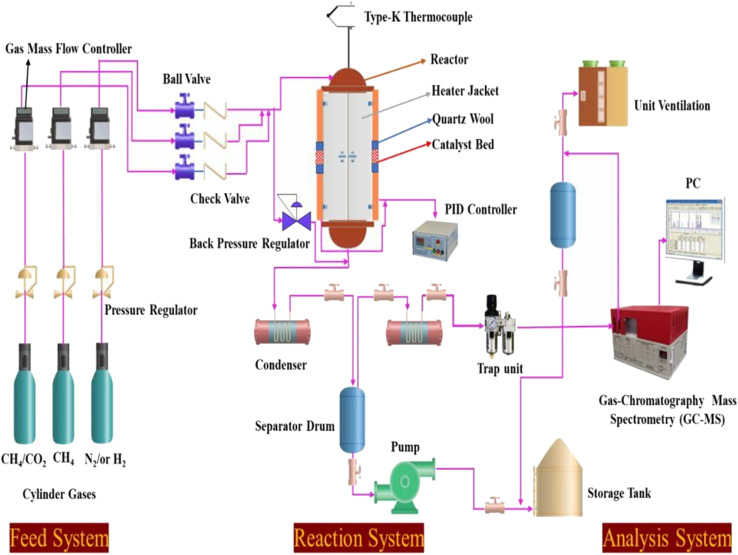
Schematic diagram of the experimental setup for the dry reforming of methane.

Catalyst tests were carried out in a stainless-steel packed-bed reactor with an internal diameter of 10 mm and a length of 300 mm, as depicted in Scheme 1. The feed gas consisted of either pure CH_4_ or 50% CH_4_/CO_2_ and its flow rate was controlled by a digital mass flow controller (MFC, Brocks Instrument) towards the reaction zones. For each run, about 300 mg of the sample (particle size 0.5 μm) was diluted with sand (particle size 0.5 μm) at the ratio of 1 : 6 and placed in the center of the reactor with quartz wool at both ends. Prior to the reaction, the catalyst was activated *in situ* at 500 °C in H_2_ flow for 1 h. Each catalyst was evaluated within a temperature range of 550–700 °C at a constant weight hourly space velocity (WHSV) of 12 000 mL g^−1^_cat_ h^−1^. The reaction temperature was controlled by embedding a type-K thermocouple inside the catalyst center. The reactions were carried out isothermally for 10 h time-on-stream. The reaction products were analyzed online every 30 min with a gas chromatograph (SRI 8610C) equipped with a flame ionized detector (GC-FID) and thermal conductivity detector (TCD) for H_2_, CO_2_, CO, H_2_O, and hydrocarbons. The effluent line of the reactor to the GC injector was kept at 110 °C to avoid the potential condensation of the hydrocarbons. From Table 1, the Ni, Mg, Fe, and Pt percentages were found to be very close to the theoretical values using ICP elemental analysis, which could be due to the incomplete precipitation of the nickel, magnesium, iron, and platinum metal precursors used during the co-impregnation process.

**Table 1:** XRD and ICP analysis of the Ni-based Al_2_O_3_-CeO_2_ composite catalysts

Catalysts	Metal loading^a^ (wt%)						*D* b (nm)
	Al	Ce	Fe	Ni	Mg	Pt	
Al_2_O_3_-CeO_2_	51.2	48.8	—	—	—	—	—
Ni/Al_2_O_3_-CeO_2_	48.1	48.0	—	3.9	—	—	36
FeNi/Al_2_O_3_-CeO_2_	47.1	46.8	2.1	4.0	—	—	32
Pt/FeNi/Al_2_O_3_-CeO_2_	47.5	46.5	2.0	3.9	—	0.005	26
MgFe/Ni/Al_2_O_3_-CeO_2_	47.0	46.8	1.9	3.8	0.5	—	33
Pt/MgFe/Ni/Al_2_O_3_-CeO_2_	47.2	46.4	2.1	3.6	0.6	0.005	27

a Determined by ICP analysis.

b Estimated by the Debye–Scherrer equation for Ni (200) of XRD.

## 3. Results and discussion

### 3.1. Characterization of the catalyst

**Fig. 1:**
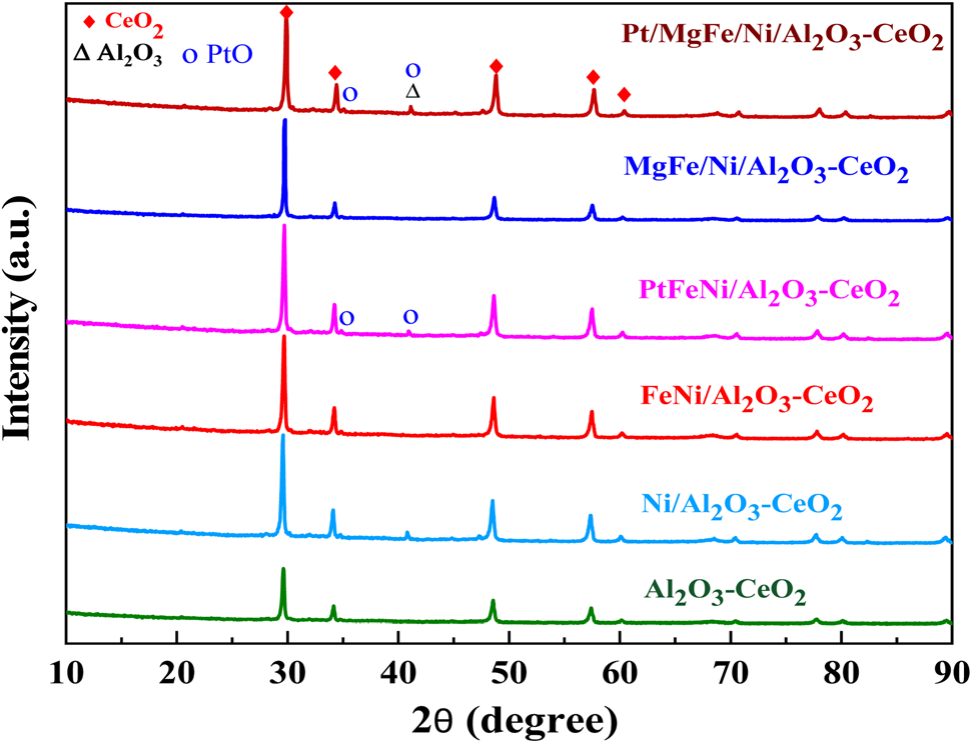
XRD patterns of Ni-based Al_2_O_3_-CeO_2_ composite catalysts.

The powder X-ray diffraction patterns for the thermally calcined Ni-based Al_2_O_3_-CeO_2_ composite catalysts with metal additives are shown in Fig. 1. The diffraction peaks observed at 2*θ* = 28, 33, 48 and 57° indicate the presence of the cubic crystal structure of the CeO_2_ support.^28^ All samples displayed diffraction peaks at 2*θ* = 38, 45, 67°, which are attributed to the γ-Al_2_O_3_ support. Peaks were barely seen at 2*θ* = 23.5, 35 and 60.5°, which can be assigned to the NiO (006), NiO (009) and NiO (110) phases, respectively. XRD signatures of MgO, Fe_2_O_3_, and Pt were not observed in the Ni-based Al_2_O_3_-CeO_2_ composite catalysts. This could be due to the presence of a small amount of oxide and also the successful incorporation of these metals into the Ni-based Al_2_O_3_-CeO_2_ structure.^29^ As listed in Table 1, elemental analysis confirmed the presence of these metals. The average crystalline size of nickel was calculated by using the Debye–Scherrer equation (Table 1). As a function of the metal oxide(s) content, the nickel crystal size decreased from 36 to 26 nm. As a result, the crystallite size also produced oxygen vacancies, in addition to the production of oxygen vacancies mediated by dopants.^30^ This phenomenon could be associated with the effects of the metal oxides and nickel that remained on the surface of the sample and inhibited the growth of Ni crystals. This was confirmed by the ICP elemental analysis results shown in Table 1.

**Fig. 2:**
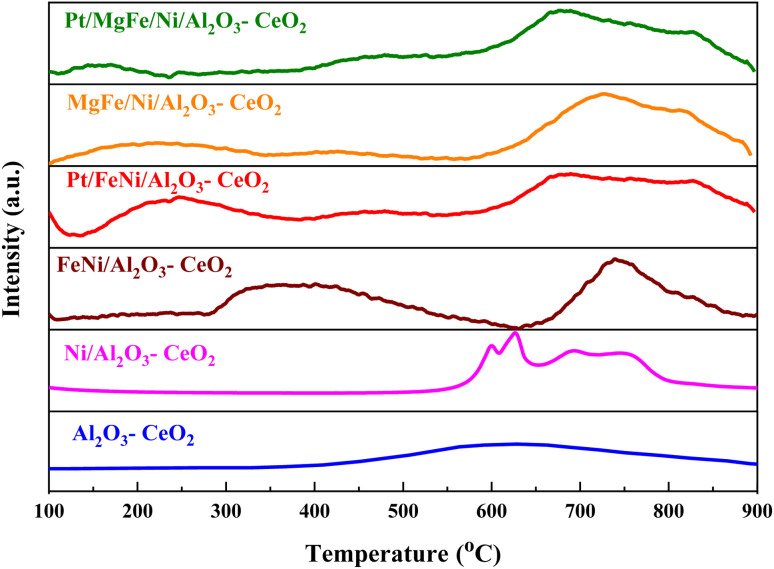
H_2_-TPR profiles of Ni-based Al_2_O_3_-CeO_2_ composite catalysts.

The H_2_-TPR profiles of the reduced Ni-based Al_2_O_3_-CeO_2_ composite catalysts showed four well-defined reduction peaks in the range of 550–850 °C (Fig. 2). These reduction peaks correspond to NiO incorporated within the structure of Al_2_O_3_-CeO_2_.^31^ The broad peak at 492–793 °C is associated with the bulk reduction of Ce^4+^ to Ce^3+^.^32^ The process of NiO reduction is thought to be a complex solid–gas reaction driven by temperature, reducing gas concentration, and the presence of additives (Mg, Fe, and Pt).^33^ This is consistent with the findings of Manukyan *et al.*^34^ The decrease began with the initial nucleation of Ni in the temperature range from (265 to 545 °C). During this phase, Ni nuclei appeared on the outer surface of NiO particles. This gradual process eventually resulted in the formation of small channels, which began to propagate and initiate the second step of the reduction. A dominant peak was formed between 565 and 900 °C, indicating the second stage of reduction on the majority of the NiO and metal oxide(s) particles. The small channels evolved into bulk NiO. Further Ni nucleation proceeded on the inner surface of these channels, resulting in the development of Ni nano-layers. The nano-layers then begin branching and interconnecting the network until the NiO phase was totally reduced to Ni.^32,34,35^ The addition of Fe and Mg metals into the Ni-based Al_2_O_3_-CeO_2_ catalyst modified the reduction process and caused the decay of the peak connected to unbound NiO, whereas the observation was made for the Fe_2_O_3_-doped systems. It should be noted that the presence of alkaline metal oxide(s) in the catalyst had a substantial impact on the reduction behavior of the NiO catalysts. In addition to the decrease in the temperature for the ceria reduction, promoted by the presence of the Fe_2_O_3_, the supplementary reduction of Fe_2_O_3_ was observed as previously reported by Wimmers *et al.*^36^ who studied the reduction of Fe_2_O_3_ and proposed a reduction in two steps, Fe_2_O_3_ → Fe_3_O_4_ → Fe, with no formation of FeO. For the same oxide, other authors proposed a three-step reduction process, which considered FeO formation dealing with Fe_2_O_3_ → Fe_3_O_4_ at about 400 °C, Fe_3_O_4_ → FeO at about 600 °C and finally, FeO → Fe at higher temperatures.^37^ Irrespective of the number of reduction steps of the Fe_2_O_3_, the separation of its reduction from that of the CeO_2_ overlapping reduction zones is hard to achieve. However, the Ni-based Al_2_O_3_-CeO_2_ catalyst without any platinum content was reduced at a much higher temperature than catalysts with platinum. The addition of platinum to Ni particles causes easily reducible NiO particles, thereby decreasing the reduction temperature because of the strong contact between Ni and Pt. Therefore, the Pt/FeNi/Al_2_O_3_-CeO_2_ and Pt/MgFe/Ni-based Al_2_O_3_-CeO_2_ catalysts allowed the reduction in the selectivity for carbon and reached values closer to equilibrium at lower reaction temperatures. This implies that the platinum interaction with the support also has a significant effect on increasing the reducibility of the support.^38^ Platinum helps reduce the oxide phases through its ability to facilitate dissociative hydrogen adsorption. Hydrogen has been observed to adsorb and dissociate on the surface of the platinum, whereby it spills over to the entire surface of the support.^7,17^ On the metallic surface, hydrogen molecules dissociate into hydrogen atoms that diffuse to the Ni^2+^ and can react with NiO, resulting in the uptake of the hydrogen. Nevertheless, the rate of reduction of NiO depends not only on its chemical nature but also on the nucleation process by which metallic nuclei are generated.^14^ Compared with FeNi/Al_2_O_3_-CeO_2_ and MgFe/Ni/Al_2_O_3_-CeO_2_ catalysts, it can be assumed that the highly dispersed and NiO in Pt/FeNi/Al_2_O_3_-CeO_2_ and Pt/MgFe/Ni/Al_2_O_3_-CeO_2_ could be more easily reduced by hydrogen atoms from the spillover effect due to the unique interactions between Ni, and Pt species and served as metallic nuclei to facilitate the reduction of Fe^2+^ and Ni^2+^. The Al_2_O_3_-CeO_2_ support plays a key role in the active site dispersion, activity and stability. To improve the reducibility, and enhance the oxygen mobility and metal dispersion, γ-Al_2_O_3_ material was modified by adding CeO_2_ and the formation of the Al_2_O_3_-CeO_2_ support. Further addition of metal oxide promoters improves the reducibility and chemisorption capacity of Ni-based Al_2_O_3_-CeO_2_ catalysts due to a better dispersion of bi/tri-metallic catalysts on the Al_2_O_3_-CeO_2_ support. The better interaction between the nickel particles and MO_*x*_-doped ceria supports could be associated with an anchoring effect inside the mesoporous structure of the support, as revealed by the textural characterization results (Fig. 5 and Table 3). It has been demonstrated that metal nanoparticles confined in mesoporous CeO_2_ enhance the metal support interaction and, therefore, the catalytic activity.^39^ Specifically, a stronger Ni-based Al_2_O_3_-CeO_2_ interaction inhibits the Ni particle agglomeration and coke deposition, which leads to better stability of the catalysts in the DRM reaction.

**Fig. 3:**
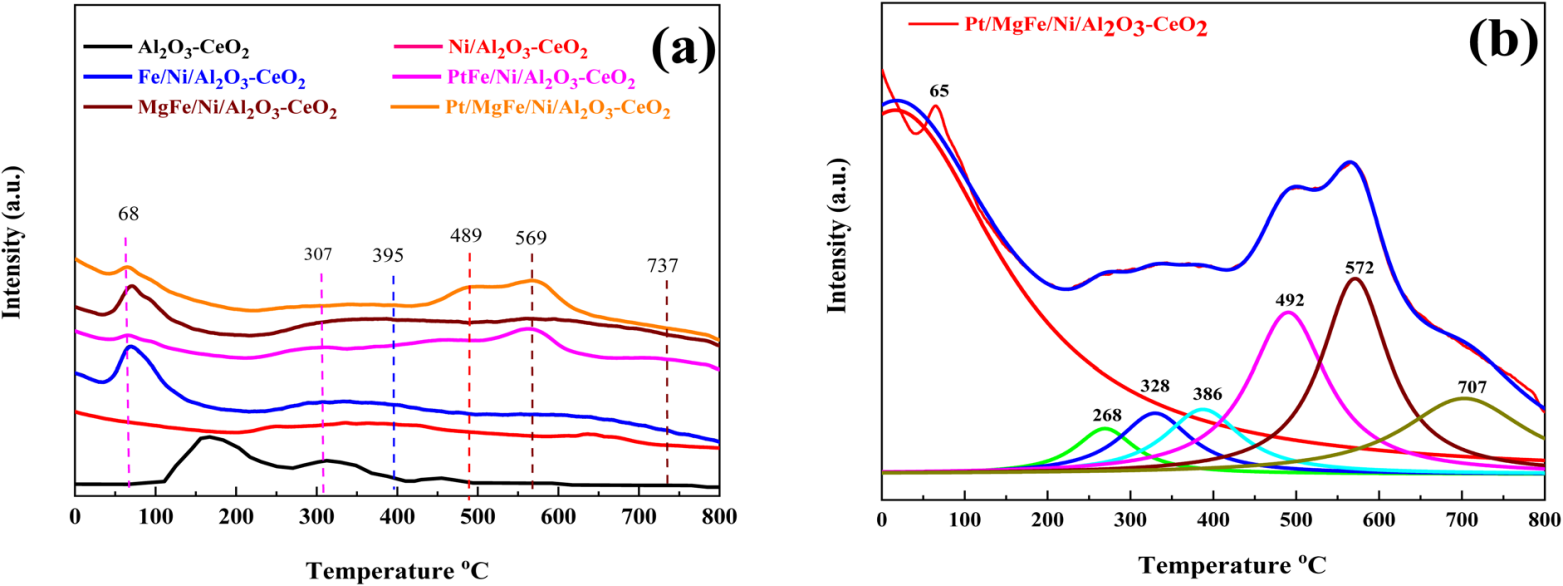
CO_2_-TPD profiles of (a) Ni-based Al_2_O_3_-CeO_2_. (b) The fine structure of the Pt/MgFe/Ni/Al_2_O_3_-CeO_2_ composite catalysts.

The number of surface alkaline centers also has an important influence on the DRM process. According to prior research, surface alkaline sites can adsorb and activate CO_2_ molecules, resulting in reactive intermediates and surface oxygen species.^40^ The adsorbed oxygen species are active in removing the initially produced carbon deposition over time, which is beneficial for the DRM process. As a result, CO_2_-TPD was employed to characterize the surface alkalinity of the catalysts. The CO_2_-TPD results are shown in Fig. 3 and changes in the weak, moderate and strongly basic sites are presented in Table 2. The CO_2_-TPD profile of the samples shows weakly basic sites between 60 and 200 °C, moderately basic sites between 200 and 400 °C, and strong sites between 400 and 800 °C, respectively.^27^ The total amount of CO_2_ was estimated from the integration of the CO_2_-TPD peak area desorbed, which obviously increased with the addition of metal oxides as compared to Ni-based Al_2_O_3_-CeO_2_. Typically, it was reported that the peak below 200 °C was attributed to the desorption of CO_2_ on the weakly alkaline sites. Due to its easy desorption, this part of adsorbed CO_2_ could have a limited contribution to the reaction. The CO_2_ adsorbed on strongly basic sites was desorbed at high temperatures.^27^ As a result, the CO_2_ desorption peak area improved significantly at higher temperatures, indicating that the amount of adsorbed/desorbed CO_2_ was increased at 550 °C, which is in the range of the reaction temperature and favorable for improving the reactivity.

**Table 2:** Summary of CO_2_-TPD of Ni-based Al_2_O_3_-CeO_2_ composite catalysts

Catalysts	CO_2_ desorption^a^ (mmol g^−1^)			
	60–200 °C	200–400 °C	400–800 °C	Total
Al_2_O_3_-CeO_2_	0.11	0.15	0.09	0.35
Ni/Al_2_O_3_-CeO_2_	—	0.34	0.11	0.45
FeNi/Al_2_O_3_-CeO_2_	0.39	0.08	0.58	1.04
Pt/FeNi/Al_2_O_3_-CeO_2_	0.03	0.26	1.25	1.54
MgFe/Ni/Al_2_O_3_-CeO_2_	0.32	0.11	0.71	1.14
Pt/MgFe/Ni/Al_2_O_3_-CeO_2_	0.08	0.30	2.15	2.53

a The amount and strength of base sites were estimated from CO_2_-TPD profiles.

Koo *et al.*^41^ reported that besides weak basic sites, moderate basic sites, and strong basic sites are favorable for depressing the coke formation in MgO-promoted Ni catalysts. In our study, the catalysts promoted with MO_*x*_ have weak, moderate and strong basic sites, whereas Ni-based Al_2_O_3_-CeO_2_ catalysts have only weak and moderate basic sites. Therefore, the intensity of TPD peaks became higher, indicating the improved CO_2_ adsorption capacity. The higher adsorption of acidic CO_2_ over the surfaces of the MO_*x*_-promoted catalysts confirmed that these catalysts are more basic as compared to Ni-based Al_2_O_3_-CeO_2_. It is well-established that the basic catalysts could improve the adsorption of CO_2_ during the DRM reaction, which supplies the surface oxygen to suppress the coke deposition. This finding is in agreement with previous studies.^41,42^ It can be concluded that the Mg^2+^-containing supports have an increased number of basic sites (greater amount of desorbed CO_2_) with respect to the support. Notably, Mg-containing zirconias showed more basic sites as compared to Ni/Al_2_O_3_-CeO_2_. Similar results over magnesia–zirconia oxides were reported by Moreno *et al.*^43^ Moreover, it was observed that Pt/FeNi/Al_2_O_3_-CeO_2_ and Pt/MgFe/Ni/Al_2_O_3_-CeO_2_ catalysts doped with Pt showed high basicity as compared to FeNi/Al_2_O_3_-CeO_2_ and MgFe/Ni/Al_2_O_3_-CeO_2_. This is attributed to the increased basicity of the catalysts, which in turn increased the rate of activation of mildly acidic CO_2_ and hence assisted in the oxidation of surface carbon and increased the catalyst resistance to deactivation.^7^ Compared to M–Ni tri/bimetallic catalysts, Pt-modified M–Ni tri/bimetallic catalysts greatly increased the contribution of CO_2_ deoxidation, and the excellent Pt deposition over M–Ni tri/bimetallic catalysts can be used to explain this, allowing oxygen to diffuse from the metal or support to the Pt^o^ in a process known as “reverse oxygen spillover”. This produces PtO and an increase in the concentration of the metal oxide(s) ions, which are the active sites for CO_2_ reduction.^44^ The PtO was detected by XRD (Fig. 1), which was proven by previous research.^45^ Eventually, the catalyst was provided with active oxygen species that suppressed carbon deposition, followed by catalyst deactivation.

Three desorption peaks centered around 395, 489, and 569 °C were also observed for all the catalysts despite the latter two peaks overlapping. The strength of the overlapped peaks was closely related to the addition of the metal oxides. These three peaks might be related to the strongly chemisorbed CO_2_. To meticulously investigate these TPD profiles, the Lorentz mathematical model was used to resolve the overlapped desorption peaks. For example, as shown in Fig. 3b, four distinct desorption peaks centered at 65, 268, 328, 386, 492, 572, and 707 °C were observed over the Pt/MgFe/Ni/Al_2_O_3_-CeO_2_ catalyst. This implies that more than one type of basic centers with different intensities existed in the mesoporous framework of the Pt/MgFe/Ni/Al_2_O_3_-CeO_2_ composite catalyst. Overall, the categories of the basic centers for Ni-based Al_2_O_3_-CeO_2_ catalysts were abundant due to their own structural features as well as the promotion of the metal oxides and this is in agreement with previously reported data.^46^

**Fig. 4:**
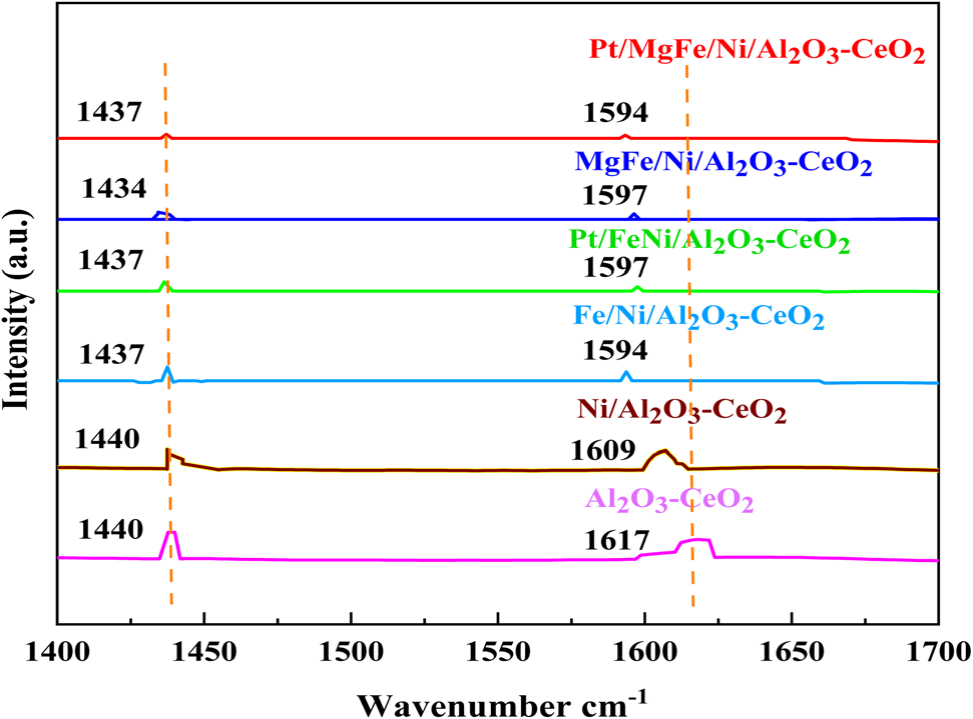
Pyridine IR spectra of Ni-based Al_2_O_3_-CeO_2_ composite catalysts.

The Brønsted and Lewis sites were found *via* the *ex situ* FTIR spectra of pyridine adsorption using a Bruker Tensor spectrophotometer. The FTIR spectra (Fig. 4 and Table 2) showed the impact of the addition of metal oxides (M = Pt, Mg, and Fe) on the Ni-based Al_2_O_3_-CeO_2_ surface acid site. One milliliter of dried pyridine was adsorbed on 30 mg of the catalyst for 14 h. The sample was dried at 120 °C for 1 h to release the loosely adsorbed pyridine from the surface of the catalyst. From that dried sample, 10 mg was taken and, to ensure homogeneity, mixed thoroughly with 200 mg of dry KBr. The mixture was then pelletized using hydraulic pressure and the FTIR spectra of the pellets were then obtained. According to Fig. 4, an absorption band at 1440 cm^−1^ or 1434 cm^−1^, which corresponds to the pyridine coordinated on Lewis acid sites on all of these catalysts, was identified in all catalysts, indicating the interaction between the pyridine and metal oxides.^47^ The bands on these catalysts at 1617 cm^−1^ and 1596 cm^−1^ were attributed to pyridinium cations generated after pyridine adsorption on Brønsted acid sites and the interactions between the adsorbed pyridine and metal oxides, respectively.^48,49^Fig. 4 and Table 3 demonstrate that the addition of metal oxides resulted in a decrease in the creation of Lewis and Brønsted acid sites, respectively, as compared to the Ni-based Al_2_O_3_-CeO_2_ catalyst. Conversely, it may be said that M–Ni-based Al_2_O_3_-CeO_2_ has greater Lewis basicity than Ni/Al_2_O_3_-CeO_2_, which is the preferred material for CO_2_ adsorption.^50^ As a result, the activity performance during the DRM is improved, and carbon deposition during formation is reduced.

**Table 3:** Results of FTIR spectra of pyridine adsorption

Catalysts	Pyridine desorption amount (mmol_Py_ g_cat_^−1^)^a^		
	1434–1440 cm^−1^	1596–1617 cm^−1^	Total
Al_2_O_3_-CeO_2_	12.991	38.069	51.060
Ni/Al_2_O_3_-CeO_2_	11.241	22.215	33.456
FeNi/Al_2_O_3_-CeO_2_	5.831	6.573	12.404
Pt/FeNi/Al_2_O_3_-CeO_2_	2.302	1.209	3.511
MgFe/Ni/Al_2_O_3_-CeO_2_	2.556	2.417	4.973
Pt/MgFe/Ni/Al_2_O_3_-CeO_2_	1.826	0.854	2.680

a The amounts were calculated by Emeis.^51^

**Fig. 5:**
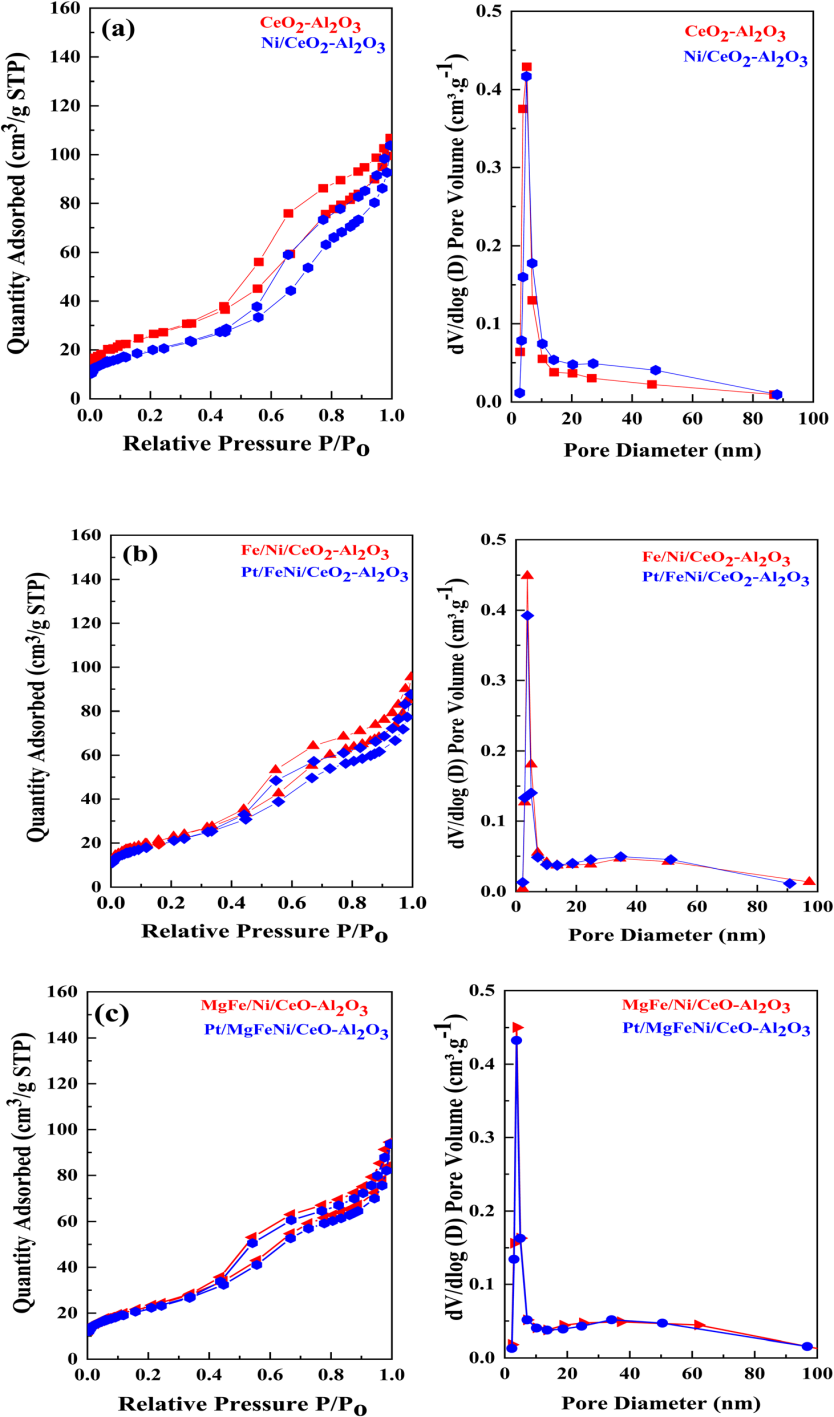
((a–c), left side) N_2_ physisorption isotherms and ((a–c), right side) BJH pore size distribution of of Ni-based Al_2_O_3_-CeO_2_ composite catalysts.

N_2_ physisorption isotherms of the as-prepared samples are shown in Fig. 5 with the corresponding pore size distribution shown as inset figures. All isotherms exhibited a combination of the type IV isotherm with the H_4_ type hysteresis loop, associated with capillary condensation, and indicated the formation of mesoporous structures in all the Ni-based Al_2_O_3_-CeO_2_ composite catalysts. The BJH method was used to estimate the pore size distributions by using the adsorption branch. Metal oxides doped Ni-based Al_2_O_2_-CeO_2_ catalysts displayed a hierarchically bi-modal porous structure with an apex of 3.8 nm (Fig. 5b) and the other between 34 and 38 nm, both of which fall within the mesoporous range; in contrast, Ni-based Al_2_O_2_-CeO_2_ and Al_2_O_2_-CeO_2_ only presented an apex. It has been established that the presence of the porous architecture in metal oxides-doped Ni-based Al_2_O_2_-CeO_2_ catalysts has a favorable influence on the improvement of catalytic activity because the connected internal voids might potentially function as efficient transport channels.^30,52^Table 4 summarizes the total surface area, micropore surface area, external surface area, mesopore volume pore size and diameter of Ni-based Al_2_O_3_-CeO_2_ composite catalysts. For comparison, the Al_2_O_3_-CeO_2_ powder was also measured. The surface areas of Al_2_O_3_-CeO_2_, Ni/Al_2_O_3_-CeO_2_ and Pt/MgFe/Ni/Al_2_O_3_-CeO_2_ were found to be 92, 86, and 76 cm^2^ g^−1^, respectively, suggesting that the addition of metal promoters reduced the total surface area. All the investigated metals influenced the textural properties of the Ni-based Al_2_O_3_-CeO_2_ catalyst but the extent was varied from metal to metal. This might result from the added metals physically blocking the support pores.^53^ The results suggest that the doped metals entered the pores of the Al_2_O_3_-CeO_2_ during the doping process, thereby affecting the mesoporosity and pore volume of the support.

**Table 4:** Physical properties of the investigated samples obtained from the nitrogen physisorption of Ni-based Al_2_O_3_-CeO_2_ composite catalysts

Catalysts	*S*_BET_ a (m^2^ g^−1^)	*S*_mico_ b (m^2^ g^−1^)	*S*_meso_ c (m^2^ g^−1^)	*V*_tot_ d (cm^3^ g^−1^)	Pore size^e^ (nm)
Al_2_O_3_-CeO_2_	92	3	89	0.04	5.59
Ni/Al_2_O_3_-CeO_2_	86	3	66	0.03	5.56
FeNi/Al_2_O_3_-CeO_2_	82	1	80	0.03	5.49
Pt/FeNi/Al_2_O_3_-CeO_2_	78	1	76	0.02	5.55
MgFe/Ni/Al_2_O_3_-CeO_2_	80	1	82	0.03	5.48
Pt/MgFe/Ni/Al_2_O_3_-CeO_2_	76	2	76	0.02	5.69

a Estimated by the Brunauer–Emmett–Teller (BET) at the *p*/*p*_o_ in the range of 0.05–0.30.

b Micropore area and micropore volumes were determined using the *t*-plot method.

c Estimated by BJH at the adsorbed amount at the *p*/*p*_o_ = 0.99 single point.

d Estimated by a *t*-plot.

e Estimated by BJH desorption average pore diameter.

### 3.2. Catalytic performance in DRM

**Fig. 6:**
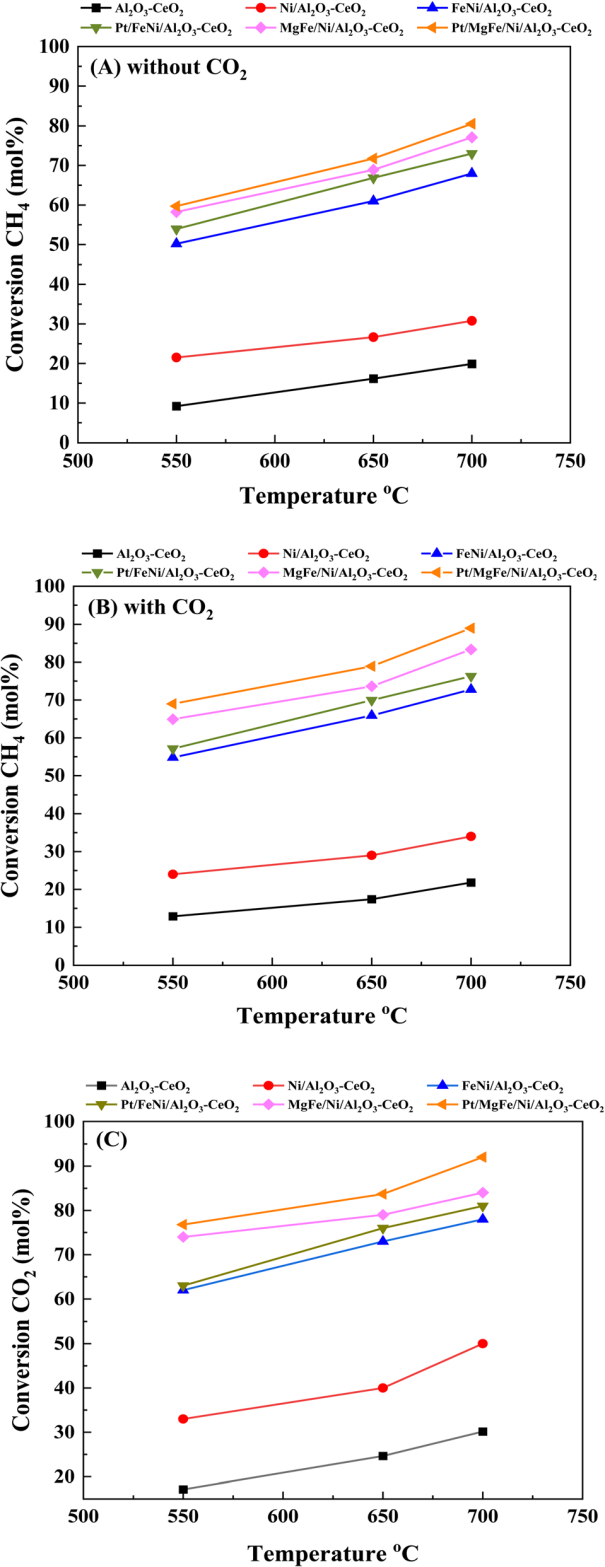
Activity of catalysts for DRM: (A) CH_4_ conversion (without CO_2_); (B) CH_4_ conversion (with CO_2_) and (C) CO_2_ conversion. Reaction conditions: reaction temperature, 550–700 °C; *P* = 1 bar; feed gas, pure CH_4_ (without CO_2_) and CH_4_/CO_2_ = 50/50 (with CO_2_), flow rate = 60 mL min^−1^, WHSV = 12 000 mL g^−1^_cat_ h^−1^.

Fig. 6(A)–(C) present catalytic results of the DRM at 550–700 °C in the presence of pure CH_4_ and a CH_4_/CO_2_ mixture (50 : 50). As shown in Fig. S1 (ESI),†Tables 5 and 6, the activity and stability of the catalysts, as well as the H_2_/CO product ratio were measured at 550–700 °C for 600 min time-on-stream. For all catalysts, the CO_2_ conversion was higher than that of CH_4_ and the conversions of both CO_2_ and CH_4_ were enhanced with increasing reaction temperature, indicating the occurrence of side reactions such as the reverse water gas shift reaction (CO_2_ + H_2_ → CO + H_2_O).^7,29^ In the absence of CO_2_ feeding (pure CH_4_), a significant amount of CO was formed over different catalysts due to the decomposition of methane (CH_4_ → C + H_2_) and a further oxidative regeneration (C + O_2_ → CO_*x*_) process.^54^ The H_2_/CO ratios were also increased as the reaction temperature increased because the RWGS reaction would gradually be prevented at elevated temperatures.^46^ The presence of CO_2_ promoted CH_4_ conversion (Fig. 6, Tables 5 and 6), catalyst stability and H_2_/CO ratio as compared to the catalysts in the absence of CO_2_ (Fig. S1†) at all reaction temperatures.

**Table 5:** Summary of the catalytic testing of Ni-based Al_2_O_3_-CeO_2_ composite catalysts

Catalysts	Reaction temp. (°C)	Conversion (mol%)		Selectivity (mol%)		H_2_/CO	DF^a^ (%)	TOF_CH_4__ (S^−1^)	TOF_CO_2__ (S^−1^)
		CH_4_	CO_2_	H_2_	CO				
Al_2_O_3_-CeO_2_	550	13	17	48	77	0.58	−132	1.21	1.42
	650	17	25	55	86	0.63	−99	1.49	1.55
	700	22	30	64	88	0.73	−114	1.54	1.77
Ni/Al_2_O_3_-CeO_2_	550	24	33	48	77	0.62	−120	1.93	2.64
	650	29	40	55	86	0.64	−103	1.98	2.77
	700	34	50	64	88	0.73	−111	2.13	2.92
FeNi/Al_2_O_3_-CeO_2_	550	54	62	60	86	0.70	−70	2.43	2.74
	650	65	73	71	90	0.79	−56	2.64	2.83
	700	71	78	80	91	0.87	−49	2.78	2.97
Pt/FeNi/Al_2_O_3_-CeO_2_	550	57	63	70	88	0.78	−65	2.76	2.96
	650	69	76	75	90	0.83	−52	2.98	3.28
	700	75	81	84	92	0.92	−46	3.18	3.52
MgFe/Ni/Al_2_O_3_-CeO_2_	550	65	74	80	96	0.82	−60	2.94	3.37
	650	74	79	85	94	0.90	−43	3.10	3.58
	700	83	84	91	95	0.96	−40	3.46	3.89
Pt/MgFe/Ni/Al_2_O_3_-CeO_2_	550	69	76	85	92	0.92	−56	3.33	3.64
	650	79	83	87	96	0.94	−42	3.63	3.96
	700	89	92	93	96	0.97	−36	3.99	4.46

a Deactivation Factor (DF) = [(final CH_4_ conversion − initial conversion CH_4_)/initial conversion of CH_4_] × 100. Reaction conditions: CH_4_/CO_2_ = 50/50, flow rate = 60 mL min^−1^, wt. cat. = 0.3 g, *P* = 1 bar for 10 h.

**Table 6:** Catalytic activity from DRM over various catalysts^a^

Catalysts	DRM reaction conditions	CH_4_ conv.%	CO_2_ conv.%	H_2_/CO	Synthesis method	Ref.
**Monometallic system**						
4%Ni/Al_2_O_3_CeO_2_(Al/Ce = 50/50)	700 °C, CH_4_/CO_2_ = 50/50, 12 000 mL g^−1^ h^−1^, 1 bar	≈34	≈50	≈0.73	Impregnation	This work
13%Ni/Al_2_O_3_CeO_2_(Al/Ce = 50/50)	700 °C, CH_4_/CO_2_ = 50/50, 180 000 mL g^−1^ h^−1^	≈44	≈58	≈0.82	One-pot	65
10%Ni/Al_2_O_3_CeO_2_(Al/Ce = 80/20)	700 °C, CH_4_/CO_2_ = 40/40, 90 000 mL g^−1^ h^−1^, 1 bar	≈40	≈50	≈0.78	Impregnation	12

**Bi-metallic system**						
4%Ni–Fe/Al_2_O_3_CeO_2_(Al/Ce = 50/50)	700 °C, CH_4_/CO_2_ = 50/50, 12 000 mL g^−1^ h^−1^, 1 bar	≈71	≈78	≈0.87	Impregnation	This work
4%Ni–Mo/Al_2_O_3_CeO_2_(Al/Ce = 50/50)	700 °C, CH_4_/CO_2_ = 50/50, 12 000 mL g^−1^ h^−1^, 1 bar	≈71	≈75	≈0.86	Impregnation	66
20%Ni–Ru/Al_2_O_3_CeO_2_(Al/Ce = 95/5)	700 °C, CH_4_/CO_2_ = 2/2, 15 000 mL g^−1^ h^−1^, 1 bar	≈88	≈84	n.a.	Impregnation	67

**Tri-metallic system**						
PtFe/4%Ni/Al_2_O_3_CeO_2_(Al/Ce = 50/50)	700 °C, CH_4_/CO_2_ = 50/50, 12 000 mL g^−1^ h^−1^, 1 bar	≈75	≈81	≈0.92	Impregnation	This work
MgFe/4%Ni/Al_2_O_3_CeO_2_(Al/Ce = 50/50)	700 °C, CH_4_/CO_2_ = 50/50, 12 000 mL g^−1^ h^−1^, 1 bar	≈83	≈84	≈0.96	Impregnation	This work
Pt/MgFe/4%Ni/Al_2_O_3_CeO_2_(Al/Ce = 50/50)	700 °C, CH_4_/CO_2_ = 50/50, 12 000 mL g^−1^ h^−1^, 1 bar	≈89	≈92	≈0.97	Impregnation	This work
Pt/FeMo/4%Ni/Al_2_O_3_CeO_2_(Al/Ce = 50/50)	700 °C, CH_4_/CO_2_ = 50/50, 12 000 mL g^−1^ h^−1^, 1 bar	≈81	≈86	≈0.91	Impregnation	66
Ni–Co–Ru/MgO-Al_2_O_3_(Mg/Al = 1/4)	760 °C, CH_4_/CO_2_ = 1/1, 111 000 mL g^−1^ h^−1^, 1 bar	≈95	≈90	n.a.	Impregnation	68
4%NiAuPt/AlMg(Al/Mg = 90/10)	700 °C, CH_4_/CO_2_ = 1/1, 60 000 mL g^−1^ h^−1^, 1 bar	77.10	85.14	n.a.	Impregnation	69
4%NiAuPt/AlCe(Al/Ce = 90/10)	700 °C, CH_4_/CO_2_ = 1/1, 60 000 mL g^−1^ h^−1^, 1 bar	79.06	86.94	n.a.	Impregnation	69

a n.a. (not available).

The catalytic stability of the catalysts was investigated over 10 h at varied temperatures (550, 650, and 700 °C) and constant mass of catalyst. Fig. S1† depicts the CH_4_, and CO_2_ conversions, as well as the H_2_/CO molar ratio as a function of time on-stream (TOS). These results confirmed the better performance of the metal oxide(s)-doped Ni/Al_2_O_3_-CeO_2_ catalyst as compared to the Ni/Al_2_O_3_-CeO_2_ catalyst. The results revealed that all catalysts had good activity and stability during 10 h on stream (TOS). It may be concluded that the carbon deposition on the surface of the catalyst was minimal, resulting in the stable conversion and H_2_/CO ratio. The small size of Ni contributes to its excellent stability. Another reason for these catalysts' high activity is the addition of metal oxides as the second active site. This finding is consistent with previous research.^30,55^ The DRM reaction was carried out over the Al_2_O_3_-CeO_2_ support (Fig. 6A–C) as a control experiment to evaluate the potential impact of Al_2_O_3_-CeO_2_. The H_2_/CO ratio was lower than the stoichiometric value for DRM (1 mol mol^−1^) for Al_2_O_3_-CeO_2,_ however, it was increased by the addition of Ni and other metal promoters. As shown in Fig. 6A–C, Ni-based Al_2_O_3_-CeO_2_ catalysts exhibited almost two-fold the CH_4_ and CO_2_ conversions as compared the Al_2_O_3_-CeO_2_ support in the temperature range investigated. Previous studies have demonstrated that the high metal dispersion led to a large number of active sites and consequently, high activity.^7,29^ At all reaction temperatures, the Ni-based Al_2_O_3_-CeO_2_ catalyst performance was further increased significantly and activity follows the trend Pt/MgFe > MgFe > PtFe > FeNi for both CH_4_ and CO_2_. The doping of Fe_2_O_3_ into the Ni-based Al_2_O_3_-CeO_2_ catalyst resulted in an almost two-fold increase for both the CH_4_ and CO_2_ conversions (Fig. 6A–C, Tables 5 and 6). Notably, the formation of CO was higher for all Fe-doped catalysts, thereby precluding the accumulated carbon deposition on nearby Ni atoms and enhancing the catalytic activity for non-oxidative methane dehydrogenation and DRM. The redox mechanism is well-defined as the Mars-van Krevelen (MvK), which consists of two reactions: the first reaction is a reduction of the catalyst *via* hydrocarbon (methane), and the second is the re-oxidization reaction of the catalyst.^56^ Doping the FeNi/Al_2_O_3_-CeO_2_ catalyst with 0.005 wt% Pt further improved the catalyst stability and maintained the H_2_/CO ratio, probably by the initial dissociation of methane (CH_4_ → CH_3_ + H) as shown in Fig. 6A–C, Tables 5 and 6. Previous studies^11,12^ also confirmed that the presence of Pt sites can also initiate the reduction of NiO by the rapid dissociation of H_2_ and then the migration of atomic H to the NiO surface by the phenomenon of hydrogen spillover, which produces a higher mobility of hydrogen on the support surface, facilitating the access to Ni particles. The Pt/FeNi/Al_2_O_3_-CeO_2_ catalyst showed more resistance to carbon formation than Ni/Al_2_O_3_-CeO_2_ and FeNi/Al_2_O_3_-CeO_2_ catalysts (Fig. 6A–C, Tables 5 and 6). As noted in Table 5, these findings have been confirmed by calculating the deactivation factor and are consistent with literature reports.^7^ A previous study by Pawelec *et al.*^57^ demonstrated that adding 0.005%Pt to the Ni catalyst leads to the generation of nanosized NiO particles, which can be readily reduced. Based on their findings, the authors ascribed the enhancement in the performance and coke resistance over the Pt-Ni catalysts to the increase in the nickel metallic dispersion caused by the intimate contact between nickel and platinum. The effect of Mg doping on the catalytic performance of the FeNi/Al_2_O_3_-CeO_2_ catalyst was also examined, as illustrated in Fig. 6A–C, Tables 5 and 6. The excellent catalytic activity and long catalytic stability were observed over a MgFe/Ni/Al_2_O_3_-CeO_2_ catalyst. As shown in the FTIR characterization, Mg doping enhanced the Lewis basicity, which is in favor of the chemisorption of CO_2_ that would accelerate the reaction, CO_2_ + C = 2CO, thus inhibiting the carbon deposition.^50,56,58^ The Pt-doped MgFe/Ni/Al_2_O_3_-CeO_2_ catalyst showed the best performance for H_2_ selectivity; it performed better than the other five catalysts in the whole process, and reached 0.97 at 650 °C as shown in Fig. 6A–C, Tables 5 and 6, while both Ni/Al_2_O_3_-CeO_2_ and Al_2_O_3_-CeO_2_ catalysts showed higher CO selectivity at 550 °C. The Pt/Mg-Fe/Ni/Al_2_O_3_-CeO_2_ showed the highest CH_4_ and CO_2_ conversions and H_2_/CO values for all the temperatures, which indicated that the cooperative interaction between metals and the support could suppress the RWGS reaction. Therefore, Pt/MgFe/Ni/Al_2_O_3_-CeO_2_ is considered a promising candidate for the DRM reaction in terms of activity, stability and selectivity. Furthermore, as summarized in Fig. 6A–C, Tables 5 and 6, the highest specific activity was obtained, which was followed by Pt/MgFe/Ni/Al_2_O_3_-CeO_2_ > MgFe/Ni/Al_2_O_3_-CeO_2_ > PtFe/Ni/Al_2_O_3_-CeO_2_ > Fe/Ni/Al_2_O_3_-CeO_2_. Surface basicity, oxygen vacancy and redox properties are crucial for enhancing the CO_2_ adsorption capacity and carbonate species formation.^59^ The addition of metal oxide(s) and rare earth elements (Ce_2_O_3_) effectively enhance the surface basicity and redox properties of the catalysts, which further affect the CO_2_ adsorption capacity of the Al_2_O_3_-CeO_2_ catalyst. The characterization analyses and catalytic tests revealed that the introduction of metal oxide(s) into the Ni/Al_2_O_3_-CeO_2_ catalyst generated more coordination unsaturated Ni atoms, oxygen vacancies, defects and active sites for the DRM reaction. The existence of Pt can initiate the NiO reduction process by the rapid dissociation of H_2_ and migration of atomic H to the NiO surface by the hydrogen spillover phenomenon, which can retrain Ni in the metallic state under DRM conditions. Niu *et al.*^60^ demonstrated that the metal with lower electronegativity enhanced CO_2_ activation, which had a positive impact on the surface oxygen concentration and promoted the oxidation of the surface carbon species, thus reducing the carbon formation and improving the catalyst stability. The addition of Pt to the catalyst stabilized the size of the Ni particles and prevented Ni from becoming encapsulated in carbon. This phenomenon occurs because of nickel particles being anchored by several carbon layers that grow in multiple directions. According to García-Diéguez *et al.*,^61^ Pt addition to the Ni/Al_2_O_3_ catalyst decreases global carbon synthesis, and the C generated in tri-metallic catalysts (Pt/M–Ni/Al_2_O_3_-CeO_2_) appears to be less tightly attached to the Ni than in the monometallic and bi-metallic Ni/Al_2_O_3_-CeO_2_ catalyst. Furthermore, unlike Ni, carbon is gasified on Pt particles rather than dispersed, resulting in the constant activity of Pt sites. According to Niu *et al.*, the electronic structure of active sites is modified in bimetallic catalysts, which affects the adsorption of specific reagents. When compared to monometallic catalysts, it reduces the activation energy of CH_4_ dissociation and CO_2_ activation. Furthermore, it increases the responsiveness of surface oxygen species, which improves carbon species suppression. The results (Table 7) show that Pt/M–Ni has lower carbon deposition than Ni due to (i) a higher energy barrier for the decomposition of the 
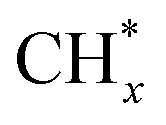
 species, which led to C* (
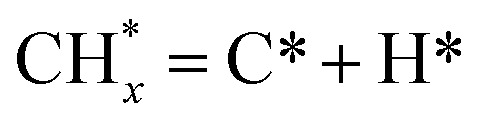
, step (5), plausible catalytic mechanism) and (ii) lower energy barriers for the oxidation of 
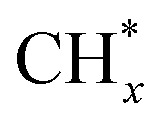
 species (
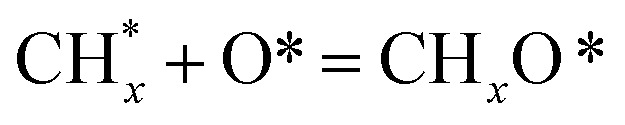
, step (17), plausible catalytic mechanism, where CH_*x*_O* is an intermediate for CO*) and C* species (C* + O* = CO* + *). As a result, the carbon concentration on the surface of Pt/M–Ni was lowered. Pt/M–Ni also weakens hydrogen dissociation, lowering the RWGS and increasing H_2_ generation. To better understand the influence of metal promoters on the activities of the M–Ni/Al_2_O_3_-CeO_2_ catalysts for the DRM, the turnover frequencies (TOFs), which reflect the intrinsic activity of the active sites, were calculated based on the Ni crystallites from XRD and the initial CH_4_ conversions (*X*_CH_4__) at 550–700 °C, and the results are listed in Table 5. For all catalysts, the TOF was improved by increasing the reaction temperature, and also by secondary and tertiary metal doping, consistent with the variation in CH_4_ conversions. These observations are in agreement with the conversion and TPR results, which revealed the best catalytic activity and the highest reducibility for Ni-based catalyst-doped metal oxides. This could be ascribed to the high interaction and dispersion of metal dopants on the catalyst surface in accordance with XRD results (Table 1). The M–Ni tri/bimetallic surface modified with Pt had a lower binding energy for H* than the monometallic Ni-based Al_2_O_3_-CeO_3_ catalyst. This demonstrates that on Pt-modified M–Ni tri/bimetallic catalysts, the surface oxidation step leading to CO production (potentially CH* + O*) became the kinetically significant phase. The electrical modification by generating the Pt-modified M–Ni tri/bimetallic surface can be attributed to the catalyst composition's significant activation energy dependence.^62^ The trend of shifting the CO stretching frequency as a result of the charge transfer between Pt and Ni fits well with the tendency of variations in the methane activation energy. Additionally, the methane activation energy tends to be similar to the O* adsorption energy, but the CO_2_ activation energy tends to be similar to the C* adsorption energy with catalyst composition. This emphasizes the relationship between the heat of C* or O* adsorption and the apparent activation energy. The activation energy of CO_2_ increases as the C* adsorption energy increases, while the activation energy of CH_4_ increases as the O* adsorption energy increases. This agrees with earlier literature and emphasizes the relationship between the heat of C* or O* adsorption and the apparent activation energy. The activation energy of CO_2_ increases as the C* adsorption energy increases, while the activation energy of CH_4_ increases as the O* adsorption energy increases. This agrees with previous literature.^63^ The results show that a minimal addition of Pt could significantly reduce the energy required to activate CO_2_. On the surface of catalysts, this will encourage the dissociation of CO_2_ into CO and O. The oxidation of surface carbon and CH_*x*_, which affects the product's H_2_/CO ratio and the rate of coke generation, is another process that is greatly aided by the presence of O*. Tri/bimetallic Pt-modified Ni-based Al_2_O_3_-CeO_3_ catalyst clusters improve catalytic activity, lower the energy barrier for CO_2_ activation, and encourage the creation of surface O* *via* CO_2_ adsorptive dissociation while lowering the activation energy for CH_4_ dissociation. Surface oxygen density may be increased by the tri/bimetallic Pt-modified Ni-based Al_2_O_3_-CeO_3_ catalysts. This is consistent with the findings of CO_2_-TPD (Fig. 3). Enhancing the catalyst's resistance to the generation of coke and extending its useful life during the reaction process is advantageous. According to H_2_-TPR investigations (Fig. 2), the presence of Pt in the Ni particles causes easily reducible NiO particles, thereby decreasing the reduction temperature because of the strong contact between Ni and Pt. As a result, the Ni–Pt matrix (alloy phase) generated with CO_2_ dissociation on the metal surface is easier to remove from the surface and subsurface oxygen. Similarly, the Ni-based Al_2_O_3_-CeO_3_ catalyst's reducibility increased and the temperature at which the NiO species were reduced dropped after the addition of metal oxides (M = Mg and Fe). This is consistent with the findings of H_2_-TPR (Fig. 2). The estimated TOF numbers are consistent with the literature findings that demonstrated higher methane conversion and syngas yield at higher TOF numbers.^64^ In general, the coke generated by methane decomposition (step (6), plausible catalytic mechanism) and the CO disproportionation reaction (step (19), plausible catalytic mechanism) in the methane dry reforming reaction may cover the metal active sites and cause the catalyst to deactivate quickly. A 10 hours long-term durability test at 700 °C was carried out to further evaluate the potential utilization of M–Ni/Al_2_O_3_-CeO_2_ catalysts. The small decrease in catalytic activity seen in Fig. S1† and Table 7 could be attributed to a change in catalyst surface during the reaction process. The M–Ni/Al_2_O_3_-CeO_2_ catalysts exhibited strong carbon resistance over a 10 hours procedure. Fig. S1† and Table 6 indicate that the M–Ni/Al_2_O_3_-CeO_2_ catalysts maintained their CH_4_ conversion, CO_2_ conversion, and H_2_/CO ratio in the presence of minimal carbon deposition. This is due to the great dispersion of Ni on the support, which prevented Ni active site agglomeration and sintering and the strong metal-support contact, which kept the Ni nanoparticle size stable during high-temperature reactions. This is a well-established H_2_-TPR profile.

**Table 7:** Summary of the weight loss of Ni-based Al_2_O_3_-CeO_2_ composite catalysts

Catalyst	Weight loss%	
	*C* a	*C* b
Al_2_O_3_-CeO_2_	4.81	3.38
Ni/Al_2_O_3_-CeO_2_	4.01	2.82
Fe/Ni/Al_2_O_3_-CeO_2_	2.94	1.97
PtFe/Ni/Al_2_O_3_-CeO_2_	1.6	1.112
MgFe/Ni/Al_2_O_3_-CeO_2_	1.2	0.79
Pt/MgFe/Ni/Al_2_O_3_-CeO_2_	0.93	0.39

a Spent catalyst in the absence of CO_2_.

b Spent catalyst in the presence of CO_2_.

### 3.3. Plausible catalytic mechanism

**Fig. 7:**
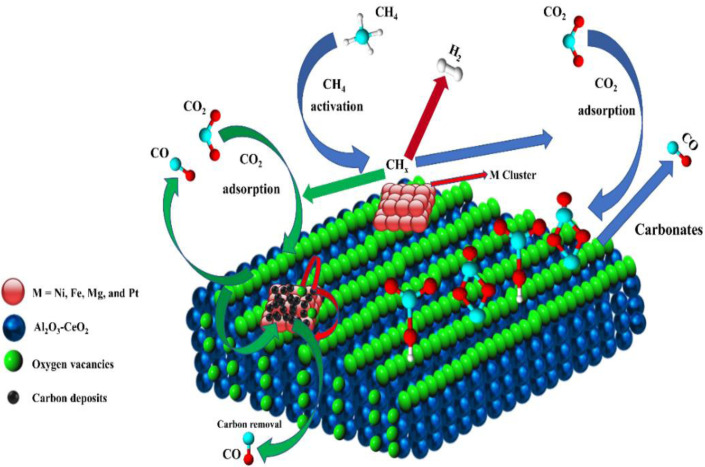
Schematic representation of a plausible reaction over the (M = Pt, Mg, and Fe) doped Ni/Al_2_O_3_-CeO_3_ catalysts.

Metal oxide(s) as promoters are crucial in the development of extremely effective DRM catalysts. All the data show that the additions had a positive effect on the surface basicity, redox characteristics, and dispersion of Ni particles, and knowing these favorable qualities can help to clarify the catalytic mechanism. The activation of CH_4_ and CO_2_ is commonly regarded as a critical step.^70^ As illustrated in Fig. 7, CH_4_ is activated over the Ni sites, and CO_2_ can be adsorbed and activated on the support surface, and the metal oxide and support interface. As demonstrated in CO_2_-TPD profiles, increasing the basicity of catalysts can increase the rate of CO_2_ activation, which has a major impact on catalytic performance (Fig. 3 and Table 2). According to our findings, the addition of metal oxides can effectively increase the surface basicity of the catalysts, thus improving the CO_2_ adsorption capacity of metal oxides (M = Pt, Mg, and Fe)-doped Ni/Al_2_O_3_-CeO_3_ catalysts. This is consistent with the *in situ* DRIFTs analysis *via* Dengsong *et al.*^71^ The adsorbed CO_2_ can form two kinds of carbonate species on the catalyst surface: bidentate carbonates and monodentate carbonates. Meanwhile, the active intermediate CH_*x*_ can react with these generated carbonates; bidentate carbonates, in particular, are more suitable for CH_*x*_ conversion. Furthermore, the produced carbonates can efficiently react with the deposited carbon; therefore the presence of the carbonates may assist in the removal of the deposited carbon, resulting in increased catalytic stability. The redox property and its effect on oxygen vacancy are crucial for improving the catalytic performance. Due to the coexistence of redox pairs, the Pt, Mg, Fe, and Ce-modified catalysts showed enhanced redox properties, thereby resulting in abundant oxygen vacancies among the M–Ni/Al_2_O_3_-CeO_3_ catalysts as shown in the H_2_-TPR profiles (Fig. 2). The abundant oxygen vacancies can provide additional active oxygen and more active sites for CO_2_ and CH_4_ activation. Active oxygen species played an essential role in reducing carbon deposition. Because surface active oxygen species can react with deposited carbon, this helps to prevent catalyst deactivation during the DRM process. Furthermore, the improved redox characteristics can enable electron transport, which can increase the rate of CH_4_/CO_2_ conversion as well as the elimination of deposited carbon. Since metal oxides were incorporated into Ni-based catalysts, the homogeneous dispersion of Ni species had a good effect on the anti-coking behavior, as shown in Table 1. The highly dispersed and small Ni nanoparticles could effectively inhibit the carbon nucleation and the subsequent growth, playing an important role in suppressing the coke formation. However, the hypothesized mechanisms for bi- and tri-metallic Ni-based catalysts are shown below for the DRM. The reaction mechanisms postulated are based on relevant compiled literature data, given as the following steps (1)–(23):1
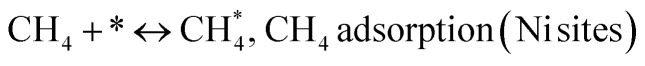
2
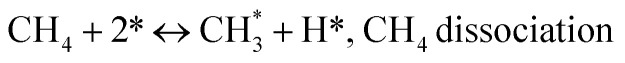
3
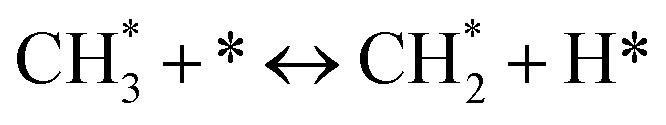
4
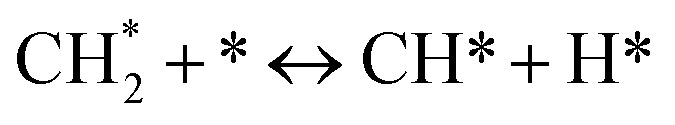

5 CH* + * ↔ C* + H*

Gathering steps (2)–(5) gives step (6):6
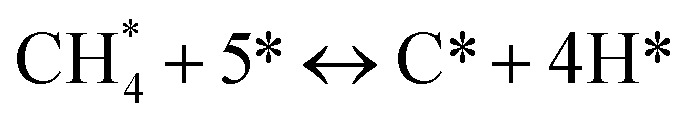
7
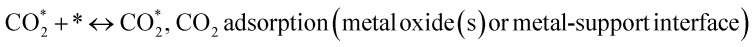
8
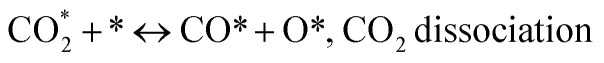
9
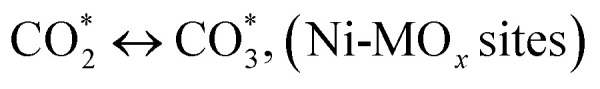
10
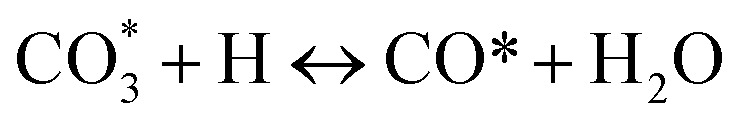
11
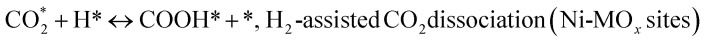

12 COOH* + * ↔ HCO* + O* (Ni-MO_*x*_ sites)

13 COOH* + * ↔ CO* + OH*, (Ni-MO_*x*_ sites)

14 HCO* + * ↔ +CO* + H*, (Ni-MO_*x*_ sites)

15
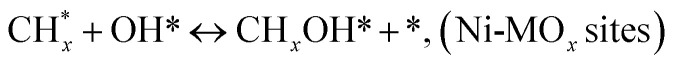

16 CH_*x*_OH* + (*x* + 1)* ↔ CO* + (*x* + 1)H*, (Ni-MO_*x*_ sites)

17
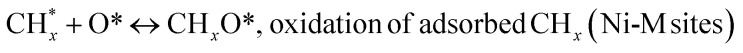

18 CH_*x*_O* + x* ↔ CO* + *x*H*, (Ni-MO_*x*_ sites)

19 C* + O_LT_ ↔ CO + O_LT_ + *, (Ni-MO_*x*_ sites)

20
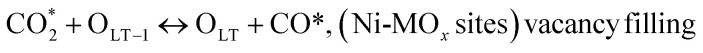

21 H_2_ + O_*x*_ ↔ O_LT−1_ + H_2_O, (Ni-MO_*x*_ sites)

22
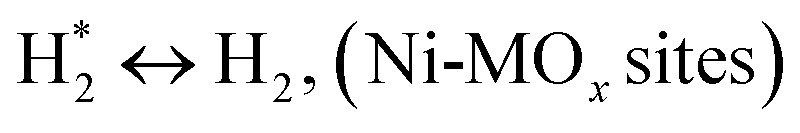

23 CO* ↔ CO, (Ni-MO_*x*_ sites)

(*) and O_LT_ represent unoccupied active sites, and lattice oxygen on the metal oxide surface, respectively. According to the above analysis, CH_4_ dissociative adsorption occurs on the Ni metal surface (steps (2)–(5)), while CO_2_ is adsorbed on the surface of the catalysts in the form of the carbonate species (step (7)). As the carbonate species encounter the methane pyrolysis products, they degrade fast into formate species, which subsequently decompose further into CO (step (9) and step (10)). CO_2_ reacts with H* generated by methane dehydrogenation to yield COOH* or HCOO* (step (11)). COOH* is subsequently decomposed into COH* and O* (step (12)) or CO* and OH* (step (13)) before COH* dehydrogenation to produce CO* and H* (step (14)). Adsorbed CH_*x*_ is oxidized *via* OH* groups to create CH_*x*_OH* (step (15)), which is then dissociated into CO* and H* (step (16)). The lattice oxygen, produced by the dissociation of CO_2_, and the facile movement of oxygen could be mobilized to the nearby Ni nanoparticles and then react with 
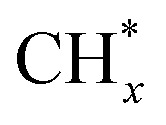
 species to give CH_*x*_O* (step (17)) and then decompose into CO* and H* (step (18)), eliminating carbon deposition and preventing catalyst deactivation. Both HCO* decomposition and 
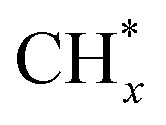
 interaction with O* result in the creation of syngas. Reaction step (19) represents solid carbon, C*, primarily from the CH_4_ molecular dissociation, which may be converted to CO *via* reacting with the lattice oxygen coming either from the CeAlO_3_ support or from metal oxide phases (M = Pt, Mg, and Fe)-doped Ni/Al_2_O_3_-CeO_3_ catalysts. Reaction step (20) represents the oxidation of the reduced CeO_2_-Al_2_O_3_ sites (O_LT-1_) by reaction with CO_2_ molecules, and step (21) represents the reduction *via*
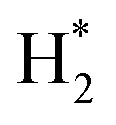
. Finally, 
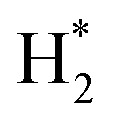
 and CO* are desorbed on the catalyst to form H_2_(g) and CO(g) (step (22) and (23)). Fan *et al.*^72^ postulated a different reaction mechanism for catalysts with basic supports, such as CeO_2_, in which CO_2_ activation occurs on the surface of the support rather than the metal active site. In this mechanism, CO_2_ is adsorbed in the vicinity of the metal particles on the catalyst support, CO_2_ (g) = CO_2_ (support), resulting in the carbonate species, CO_2_ (support) + O^2−^ = CO_3_^2−^ (support). The hydrogen is then used to reduce the carbonate (CO_3_^2−^ (support) + 2H = HCO_2_^−^ (support) + OH^−^) to produce CO.^73^ According to the theory, CO_2_ is adsorbed on promoters like Ce or CeO and dissociates into CO and O (step (5)). Following this, carbon from the decomposition of CH_4_ is deposited on the catalyst's active sites where it reacts with the adsorbed oxygen on the promoter to generate CO (O* + C* → CO*).

## 4. Conclusion

Pt, Fe, and Mg were doped into Ni-based Al_2_O_3_-CeO_2_ composite catalysts *via* an impregnation method and evaluated for DRM reaction. All doped Ni-based Al_2_O_3_-CeO_2_ composite catalysts presented significantly higher activity and H_2_ selectivity as compared to the Ni-based Al_2_O_3_-CeO_2_ composite catalysts. Characterization results showed that compared to unmodified catalysts, the catalysts treated with metal oxide (Mg, Fe, and Pt) particles had a significant number of basic sites for enhancing CO_2_ adsorption. The most stable and efficient conversion of CO_2_ and CH_4_ was achieved by the modified catalysts because they displayed the highest Ni particle dispersion, which can effectively stop the migration of Ni species. Additionally, as shown by the H_2_-TPR profile, the co-doped catalysts displayed high levels of redox cycling, which enhanced the generated oxygen vacancies and helped eliminate carbon deposition. Although all metal-doped Ni-based Al_2_O_3_-CeO_2_ catalysts showed slightly lower surface areas than the Ni/Al_2_O_3_-CeO_2_ catalyst and Al_2_O_3_-CeO_2_ support, they presented significantly higher activity and H_2_ selectivity compared to Ni-based Al_2_O_3_-CeO_2_ composite catalysts. Catalytic activity results revealed the impact of secondary and tertiary metal doping on the Ni-based Al_2_O_3_-CeO_2_ catalyst and the highest methane (>85%) and CO_2_ (∼90%) conversions and high selectivity towards H_2_/CO ratio (0.97) were obtained over the Pt/MgFe/Ni/Al_2_O_3_-CeO_2_ composite catalyst. The order of activity of the catalysts, based on the turnover frequencies, was Pt/MgFe/Ni/Al_2_O_3_-CeO_2_ > MgFe/Ni/Al_2_O_3_-CeO_2_ > Pt/FeNi/Al_2_O_3_-CeO_2_ > FeNi/Al_2_O_3_-CeO_2_ > Ni/Al_2_O_3_-CeO_2_. The observed better Pt/MgFe/Ni/Al_2_O_3_-CeO_2_ stability could be due to favorable changes in the distribution of surface basic sites, and better Ni dispersion.

## Data availability

All data generated or analyzed during this study are included in this published article in the main manuscript and ESI.†

## Conflicts of interest

The authors declare that they have no known competing financial interests or personal relationships that could have appeared to influence the work reported in this paper.

## Supplementary Material

RA-013-D3RA04809H-s001
