# Supplementary material for: The effects of Fe, Mg, and Pt-doping on the improvement of Ni stabilized on Al2O3-CeO3 catalysts for methane dry reforming
Source: RSC Adv. 2023 Nov 9;13(47):33129–45. doi: 10.1039/d3ra04809h (PMC10634349; doi:10.1039/d3ra04809h)

Electronic Supplementary Material (ESI) for RSC Advances.
This journal is © The Royal Society of Chemistry 2023

Supporting Infromation

Improvement Ni Stabilised on Al_2O_3-CeO_2 Catalysts for Dry Reforming of Methane: Effect of Fe, Mg and Pt Doping

Abbas Jawad[1,2]

ajd5d@mst.edu; abbasajd5d@gmail.com

[1]Department of Chemical & Biochemical Engineering, Missouri University of Science and Technology, 1101 N. State Street, Rolla, Missouri 65409, United States

[2]Midland Refineries Company MRC/Daura Refineries/southeastern part of Baghdad, Iraq

Figure S1: Catalytic performance of the (A) Al_2O_3-CeO_2; (B) Ni/Al_2O_3-CeO_2; (C) FeNi/Al_2O_3-CeO_2; (D) Pt/FeNi/Al_2O_3-CeO_2; (E) Mg/FeNi/Al_2O_3-CeO_2; and (F) Pt/Mg/FeNi/Al_2O_3-CeO_2 composite catalysts. Reaction condition ; reaction temperature 550-700 °C ; P = 1 bar; feed gas pure CH_4 and CH_4/CO_2 = 50/50, flow rate = 60 mL min^{-1}, WHSV = 12,000 mL min^{-1} g_{cat}^{-1}.

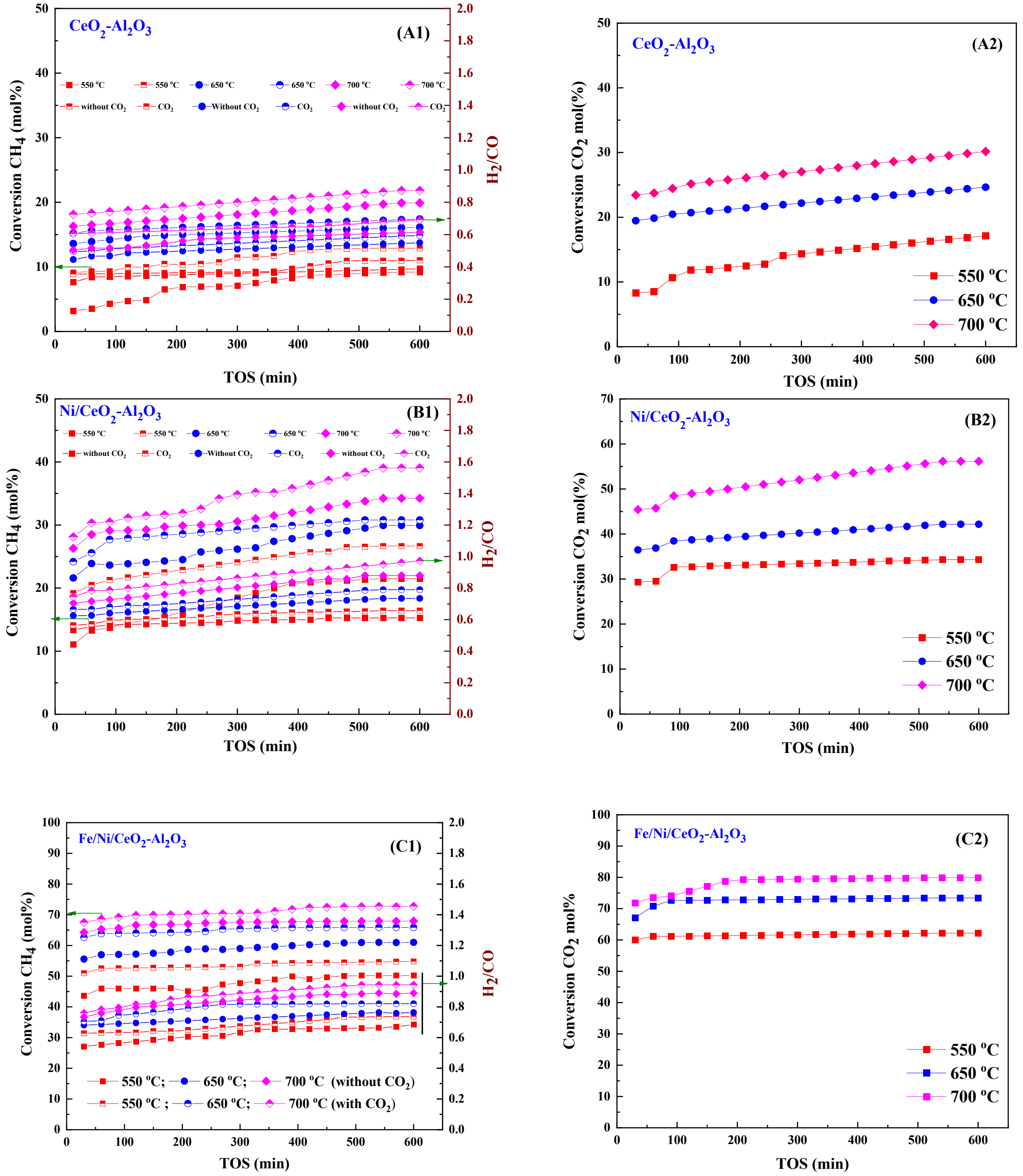

Figure S1: Catalytic performance of the (A) Al_2O_3-CeO_2; (B) Ni/Al_2O_3-CeO_2; (C) FeNi/Al_2O_3-CeO_2; (D) Pt/FeNi/Al_2O_3-CeO_2; (E) Mg/FeNi/Al_2O_3-CeO_2; and (F) Pt/Mg/FeNi/Al_2O_3-CeO_2

composite catalysts. Reaction condition ; reaction temperature 550-700 °C ; P = 1 bar; feed gas pure CH_4 and CH_4/CO_2 = 50/50, flow rate = 60 mL min^{-1}, WHSV = 12,000 mL min^{-1} g_{cat}^{-1}.

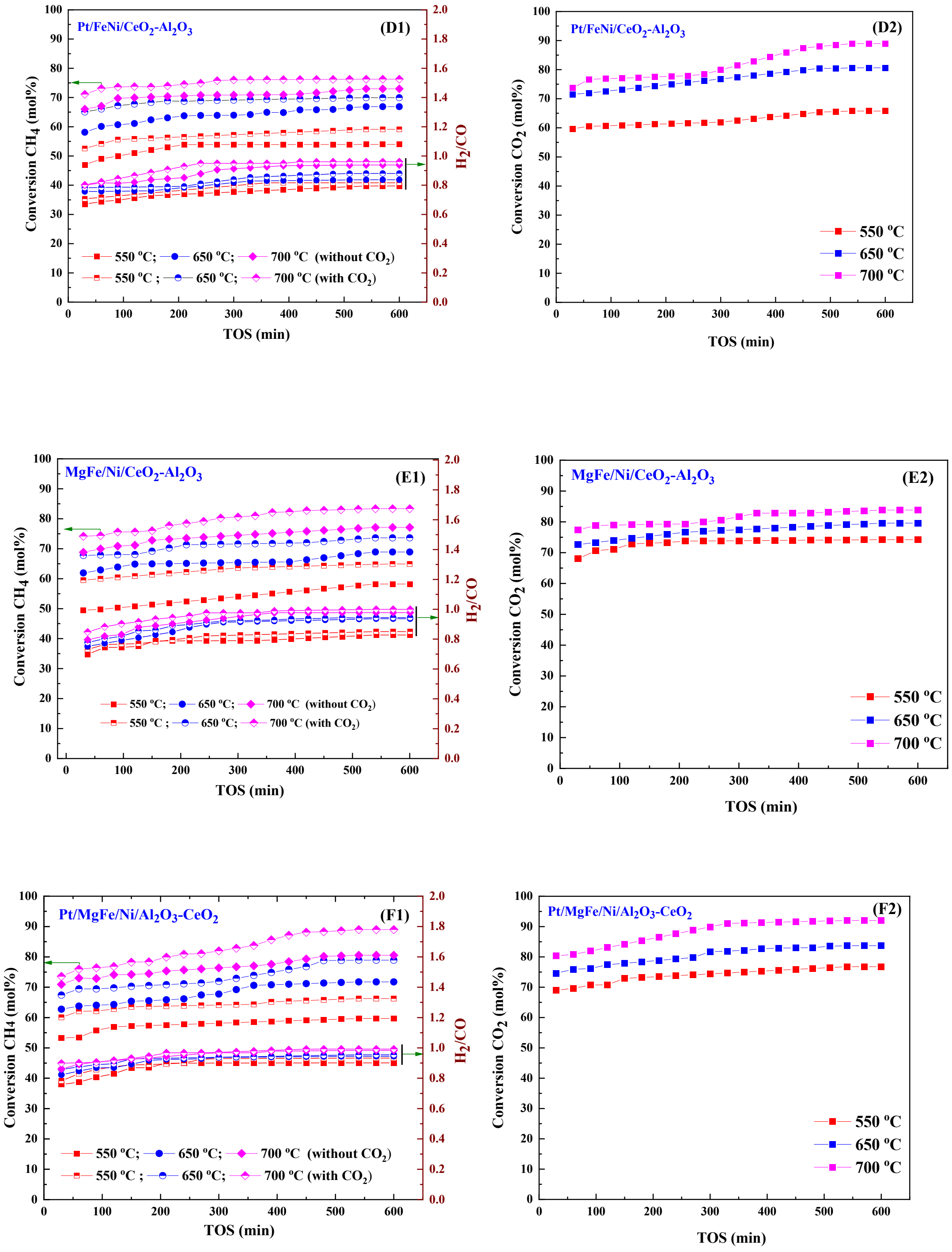

Supplement: RA-013-D3RA04809H-s001 [file RA-013-D3RA04809H-s001.pdf]
